# Bridgework—Fixed and Removable

**Published:** 1922-04

**Authors:** E. P. R. Ryan

**Affiliations:** New York City


### BRIDGEWORK—FIXED AND REMOVABLE

BY E. P. R. RYAN, D.D.S., NEW YORK CITY

The discussion of this subject, you will notice, is not fixed versus removable bridgework, but a treatise on both as practiced today by general practitioners of dentistry. The contentions and discussions in the voluminous articles of our magazines, emanating from the confirmed fixed bridge advocate, as a champion of this phase, versus the promulgators of various removable bridge systems, savors of such bias and antagonism that the general practitioner is bewildered, rather than helped, to arrive at a practical logical solution. Their conclusions are often worthless because each group is comparing its best and most modern effort with the unfortunate failure of the other system in the hands of an incapable operator.

The controversy that seems to be assuming such proportions in regard to fixed and removable bridgework is not a question of either being all right and the other all wrong in every case; it is a question of developing a system or combination of systems that will meet the requirements in each case in the nearest normal and ideal manner. The requirements of the individual case *must* be met.

Efficiency of the general practitioner today must include a knowledge of and ability in prosthetic dentistry, bridgework of all kinds, periodontoclasia, pyorrhea alveolaris, root canal technique, radiography and orthodontia. He can be successful in none unless he knows the possibilities and limitations of all.

In presenting this subject of restoration, we will classify, for comparative analysis, fixed bridgework, saddle bridges and removable bridgework, with their respective advantages and disadvantages. The physical and fundamental requirements of each class are similar and the demands upon the tissues practically the same and we shall deal with these conditions primarily.

The procedure of the examination to determine the form, class, system or style of restoration, should include the following: First, survey of the aesthetic demands; second, notation of the condition of the mucous membranes and gum tissues, and the general appearance of mouth; third, the study of condition and position, regularity or irregularity of the teeth, especially those to be used as abutments and their opposing teeth; fourth, full radiographic examination, especially of the region that is to receive the restoration; fifth, impressions and bite of the regions to be restored, subsequently mounted for study and observation. The nicety of judgment formed by this process makes for or against the success or failure of any restoration, fixed or removable. No restoration or any other procedure is worth doing which is not carefully planned and thoroughly outlined before any step is initiated.

The study of the irregularity that may be present is subsequently continued with the mounted models, notation made of the position and relationship of the teeth and the opposing cusps, and especial attention given to the planes and articulating cusps of the teeth in opposition to the contemplated bridge. Should a patient present a case of periodontoclasia, the case should be cared for. By all means, do not ignore it.

Teeth out of line, malposed or tipped should not be used by building them up into occlusion. If any dependency for the permanent result is to be had, they should be regulated and placed in as near normal position as possible before an attempt is made to insert the case. One of the greatest causes of failure in bridgework today is the use of malposed teeth built up with improper occluding planes, with the stress of mastication pounding on the sides of the roots. A leading orthodontist recently remarked: "The cause of failure of most fixed bridges is the exaggerated occlusal surfaces made to fill up spaces, without any regard to proper occluding planes which, with fixation, breaks down the peridental membrane." This error is too frequently found in all forms of bridgework, fixed, removable and saddle cases, and should always be borne in mind. We are inclined to overload the occlusion in our desire for masticating surface.

The human arch presents three classes of teeth, differing in the formation of their cusps, crowns and surfaces, and with each class the shape and form of the roots have been so arranged by nature to perform their service efficiently and successfully, with the normal amount of strain placed upon each class.

The radiographic examination of the entire mouth is not always essential, but films of the teeth you expect to carry abutments and the intervening bone tissue should always be made. You may find an unlooked for condition, a devitalized or abscessed tooth with a faulty root canal filling. Upon close examination of the conditions may alter even the type, form or system of restoration one may be inclined to apply. Know that your foundation is stable, healthy and firm, and that you are not blindly submitting your case to possible wreck by an assumption of the proper conditions of these teeth. A wreck is just as depressing with the most beautiful and scientifically built removable bridge as with a fixed piece, no matter for what reason they may fail.

A more or less common and erroneous method of examining a radiographic film consists of holding it to the light and with a casual glance at the region of the apices of the roots; if no radiolucent area is present, the film is not further scrutinized. This procedure indicates a lack of knowledge of the X-ray and a greater lack of understanding of the histological aspect of the teeth and tissues shown. Previous to any apparent degenerative change at the apex or margin of the root, a distinct symptom of pathology occurs in the bone tissue of this region. The normal, spongy, irregular bone cells take on a regular formation and this is visible in a good radiograph long before any other evidence of pulp involvement. A small hand magnifying glass is of distinct assistance in the reading of radiographs, especially in determining the condition of the peridental membrane.

Bridgework of various forms and its application renders consideration of the peridental membrane, excepting the pulp, the most important and of greatest concern because there are more failures as a result of injury to and a diseased condition of this tissue than from apical infection. This statement is open to argument, however, by those who believe that the peridental membrane is infected by the progress of pulp infection and vice versa.

In the construction of gold crowns or bands which we insert to various depths under the free margin of the gum, we overlook the fact that the peridental membrane does not stop at the alveolar margin, but that it penetrates up into the submucous layer of the gum tissue which it supports and extends into the tissues of the interproximal spaces. Noyes says: "It makes no difference how perfect a crown may be or how perfectly any damage which may have occurred to it may have been restored, unless the peridental membrane is in a healthy and fairly normal condition the tooth will be useless and the individual would be more comfortable without it."

Liberal comment on this tissue is made because in selecting bridgework, its protection from injury and misuse is of paramount importance. This membrane should receive just as serious consideration as the pulp. In the construction of restorations, many operators who hold in horror the destruction of the pulp will unwittedly injure and lacerate this membrane by cementing a malfitting and improperly contoured band around the tooth and absolutely misapply the force and stress reflected upon this tissue.

Briefly, let us consider what occurs when force is misapplied. The tooth is squeezed out of normal position; the fibres of the peridental membrane, which are intertwined by profuse numbers of blood vessels, are stretched to allow the movement and in this position the blood supply is altered; the vessels are closed at one point by the fibres and in another the vessels are dilated, with the result that the shutting off of the blood supply sets up an acute inflammatory process in this area.

It requires a very skillful operator to remove enamel and properly fit a band without injury to the peridental membrane, and unfortunately this tissue has not the power to function and recuperate which we find in the gum tissues. Few operators in making bands have the slightest idea of contouring for support of the gum tissues and disregard the necessity of restoring the normal tension on the free margin by proper shape of the cap or band. This fault is evidenced by the sluggish, turgid, abnormal tissue around the margins of many so-called good fitting bands.

The use of gold bands in bridgework, as made in the vast percentage of cases, is unnecessary. They are unsafe, unscientific and inefficient. The claim that a root which is ground down to the gum margin, or approximately so, can not be protected from splitting otherwise is erroneous. of roots and tissue shown, the radiographic examination Cast base caps, with a proper contour, made to extend under the free margin as far as safety and stress demand, will protect the root in a vastly more efficient manner and furnish protection to the peridental membrane. The class of vital teeth that are being crowned may be restored with more safety to the pulp and other tissues with proper fitting cast gold inlays.

Fixed bridges extending from one class of teeth to the other are, in a very few cases, satisfactory with any form of abutments, because cemented in place they restrict the natural movement of these teeth. For instance, a bridge extended from the cuspid, a tooth of prehension, to the first molar, a triturating tooth, denies each the individual motion and thereby eliminates stimulation to the surrounding tissue of the roots—a natural result of the masticating stress.

Bridges with cast gold inlays, three-quarter crowns and cast gold bases, which can be made sanitary, I mean in fact, not in theory, with a pin rest or extension in one terminal fitting or inlay or made with a dovetail double casting which fits into the free abutment inlay, allowing the breaking of stress, are, in the writer's practice and opinion, the only safe and acceptable fixed bridges. The number of dummy teeth supplied, however, ordinarily can not exceed two, so the limitation of any fixed bridgework is well defined.

The pinlay method of restoration for the same reason, the limitation of supplying more than two lost teeth, also is far from a universal system. This system also involves the loss of tooth structure to accommodate two or more pins and the writer's experience has been that this seriously jeopardizes the integrity of the pulp.

Let us consider the saddle bridge or restorations which are made with gold saddles having clasps for attachments. These bridges, with saddles cast in such shape as to properly distribute the force of mastication over the edentulous region and made with clasps that fit the teeth to which they attach, have an important place in the choice of restoration. The same care and treatment of the gum tissue should be followed that we use prior to the impressions for removable bridgework. The technique of construction of these bridges is well known and while there has been fevered discussion of the merits of the cast and swedged clasps, respectively, the experience of the writer in using cast clasps has been sad and disappointing. The cast gold clasp which is made firm by the small percentage of platinum grips the tooth in an unyielding manner, and even relief by polishing does not suffice when the bridge begins to settle.

It is oftentimes embarrassing to explain to a patient that the extreme looseness of a clasped tooth is of no consequence, especially when you fear upon removal that the tooth may be comfortably reposed in your clasp. For this reason, the writer changed some of these bridges and replaced the clasp with a bent contoured form which is more springy and allows some movement. The bridges of the style which have been inserted are being made under close observation, as there is little confidence in their permanency. They were, in most instances, inserted at the demand of patients who were disinclined to have cavities cut into sound teeth.

A new form of attachment is being used with this form of saddle bridgework, which is an alteration of the Roach. It consists of a sleeve or slot made to receive a flat, round, head part that is soldered to the inlay, the sleeves or slots being paralleled and soldered to the saddle. The writer sees no use of this form of attachment except in application to partial plates. The advisability of using any form of an extension upon a tooth which can only serve as a pump handle leverage is not admitted.

The removable bridge is the result of years of development and experience of all forms of prosthesis, the fundamental scheme being founded upon the success and development of inlays, crowns, fixed bridgework, plates and all manner of dentures. Many are familiar with the style and system which has been developed to such a fine degree by Dr. Peeso, and I shall but briefly deal with the technique and give the result of my experience with this system.

The making of bands for the telescope crowns and blind caps with their platinum pins, necessitates the removal of the pulp, which fact carries its own argument. The great sacrifice of tooth structure necessitated for the application of bands dealt with previously in this paper is another argument against this system. The manipulation of the platinum saddles is, in the average operator's hands, extremely difficult while making your impressions and assembling. The argument advanced that the platinum takes more kindly to the gum tissues, is entirely outweighed by the irritating effect of the unyielding position in which the saddle is held. These bridges, when snapped into place, are the acme of fit and the crowns are absolutely unyielding. The result is irritation and inflammation of the gum tissue because of the constant pressure, and is a source of trouble to the operator and the patient. This constant pressure, if not borne by the gum tissue and substructure, must then exert itself upon the abutment teeth. So, after using this system with these observations, the writer came to the conclusion that this form of bridgework was an advantage over fixed bridgework only in the fact that it was removable.

The system of the split bar and pin, which was used for a short time only, presented the same objections and the results were no more satisfactory. The advocacy of the use of saddles in removable bridges is influenced by the fact that, upon the removal of well fitting plates or saddles from edentulous mouths, we almost invariably find a good healthy pink condition of the gum tissue. This is due to the fact that intermittent pressure exercises this tissue and keeps up a good coursing blood supply in these parts.

The Chayes' system of movable removable bridgework has secured for the writer the greatest advantages, overcome the greatest objections and given to the patient the most comfortable and efficient service. Dr. Chayes' has developed several attachments that are simple, strong and efficient. The application of these attachments, in the hands of a careful operator, will meet all of the following requirements of any restoration: Elimination of the useless extirpation of pulps, the conservation of tooth structure, elimination of clasps, bands and extensions, prevention of irritation of peridental membrane, giving abutment teeth individual motion and preserving their integrity, reduction to the minimum of strain on abutment teeth, allowance of intermittent pressure to all saddles, removal and replacement by the patient with the added feature that a patient may readily regulate the bridge unassisted, and the application of all aesthetic requirements. Briefly, these attachments consist of small platinum boxes or receptacles which are termed the female part or jacket and a T-shaped part of heavy platinum, doubled upon itself, termed the male part, which perfectly fits into the former. These attachments are made in different sizes, are called bucco-lingual attachments and are easily inserted in inlays, in the teeth of all three classes.

The movement of the bridge is secured by arching the sides of the male part so that when inserted into the female part, we have the curves tangent to the straight line sides of the box. Determination of the amount of movement desired is controlled by this corresponding degree of arching.

There are several other forms of attachments which Dr. Chayes' has ingenuously devised. The idea has gained credence among many of the dental profession that this system is too technical and out of the bounds of possibility for the average practitioner. I want to emphatically contradict this statement by saying that any good dentist who properly prepares cavities for gold inlays and is efficient in casting, can, with an acquired knowledge of technique, successfully construct these bridges and manipulate this system.

The greatest percentage of abutments are made on gold inlays with practically no divergence from the typical Black cavity preparation.

It gives one the greatest pleasure to insert a movable removable bridge in the mouth of a patient and to see the satisfaction, comfort and efficiency of this substitute which is of vital importance to him and his health.

In conclusion, let me state I have given my results and conclusions of years of honest and industrious application, and that I have done this without regard to personalities or prejudices to any critic, sponsor or promulgator of any class of bridgework. We have a great duty to perform for our patients, as well as ourselves, and humanity is deserving of the good in everything and we can attain no greater success than to give each and every case the utmost of effort, science, engineering, workmanship, art and service.—*The Military Dental Journal.*

